# Home initiation of chronic non-invasive ventilation in COPD patients with chronic hypercapnic respiratory failure: a randomised controlled trial

**DOI:** 10.1136/thoraxjnl-2019-213303

**Published:** 2019-09-04

**Authors:** Marieke L Duiverman, Judith M Vonk, Gerrie Bladder, Joost P van Melle, Jellie Nieuwenhuis, Anda Hazenberg, Huib A M Kerstjens, Job F M van Boven, Peter J Wijkstra

**Affiliations:** 1 Department of Pulmonary Diseases/Home Mechanical Ventilation, University of Groningen, University Medical Center Groningen, Groningen, The Netherlands; 2 Groningen Research Institute for Asthma and COPD (GRIAC), University of Groningen, University Medical Center Groningen, Groningen, The Netherlands; 3 Department of Epidemiology, University of Groningen, University Medical Center Groningen, Groningen, The Netherlands; 4 Thoraxcenter, Department of Cardiology, University of Groningen, University Medical Center Groningen, Groningen, The Netherlands; 5 Department of General Practice & Elderly Care Medicine, University of Groningen, University Medical Center Groningen, Groningen, The Netherlands

**Keywords:** non-invasive ventilation, COPD, telemedicine

## Abstract

**Introduction:**

Chronic non-invasive ventilation (NIV) has become evidence-based care for stable hypercapnic COPD patients. While the number of patients increases, home initiation of NIV would greatly alleviate the healthcare burden. We hypothesise that home initiation of NIV with the use of telemedicine in stable hypercapnic COPD is non-inferior to in-hospital NIV initiation.

**Methods:**

Sixty-seven stable hypercapnic COPD patients were randomised to initiation of NIV in the hospital or at home using telemedicine. Primary outcome was daytime arterial carbon dioxide pressure (PaCO_2_) reduction after 6 months NIV, with a non-inferiority margin of 0.4 kPa. Secondary outcomes were health-related quality of life (HRQoL) and costs.

**Results:**

Home NIV initiation was non-inferior to in-hospital initiation (adjusted mean difference in PaCO_2_ change home vs in-hospital: 0.04 kPa (95% CI −0.31 to 0.38 kPa), with both groups showing a PaCO_2_ reduction at 6 months compared with baseline (home: from 7.3±0.9 to 6.4±0.8 kPa (p<0.001) and in-hospital: from 7.4±1.0 to 6.4±0.6 kPa (p<0.001)). In both groups, HRQoL improved without a difference in change between groups (Clinical COPD Questionnaire total score-adjusted mean difference 0.0 (95% CI −0.4 to 0.5)). Furthermore, home NIV initiation was significantly cheaper (home: median €3768 (IQR €3546–€4163) vs in-hospital: median €8537 (IQR €7540–€9175); p<0.001).

**Discussion:**

This is the first study showing that home initiation of chronic NIV in stable hypercapnic COPD patients, with the use of telemedicine, is non-inferior to in-hospital initiation, safe and reduces costs by over 50%.

**Trial registration number:**

NCT02652559.

**Key messages**

**What is the key question?**

- Is home initiation of non-invasive ventilation (NIV) in stable hypercapnic COPD non-inferior to in-hospital NIV initiation?

**What is the bottom line?**

- Home initiation of chronic NIV in stable hypercapnic COPD patients is non-inferior to in-hospital initiation, safe and saves over 50% of the costs.

**Why read on?**

- This is the first randomised controlled trial showing that home initiation of chronic NIV in COPD patients with chronic hypercapnic respiratory failure is non-inferior to hospital initiation, and is associated with savings of over 50% of the costs.

## Introduction

Long-term non-invasive ventilation (NIV) has long been controversial in patients with stable Chronic Obstructive Pulmonary Disease (COPD) and chronic hypercapnic respiratory failure (CHRF).^1–7^ However, with the introduction of high-intensity NIV, defined as a mode of ventilation with higher inspiratory pressures and a higher backup respiratory rate (BURR) aimed at a more controlled form of ventilation and improvement in gas exchange,^8 9^ clinically relevant improvements have also been shown in COPD, without undue loss of patient comfort.^10–17^ On the basis of these positive results, chronic NIV set up to target substantial arterial carbon dioxide reduction has become the standard of care for patients with severe stable COPD and CHRF.

Initiation of high-intensity NIV is a delicate process of achieving sufficient ventilatory support while keeping patients comfortable and avoiding side effects. Historically, it has been believed that initiation and titration of chronic NIV targeted at a substantial arterial carbon dioxide reduction requires a hospital admission.^10 16^ Since the number of patients with COPD, who will be initiated on chronic NIV, in the Netherlands is expected to rise in the coming years, so will the burden on our healthcare system. For instance, at our centre, the number of COPD patients initiated on chronic NIV increased from 10 in 2015 (7% of the total number of patients initiated on chronic NIV) to 76 in 2017 (33% of the total number of patients initiated on chronic NIV).

Home initiation of NIV would greatly alleviate the burden on the healthcare system and would prevent demanding hospital visits in a disabled dyspnoeic patient population, but needs to be proven safe, effective and cost-effective. In patients with neuromuscular disease, it has been shown that initiation of chronic NIV at home, with the use of telemonitoring, is non-inferior to initiation in the hospital.^18^ However, neuromuscular patients are initiated on NIV with lower ventilator settings, and therefore might adapt more easily to their NIV. We hypothesised that with extensive possibilities for remote monitoring of ventilator parameters and transcutaneous carbon dioxide (PtCO_2_) using telemedicine, home initiation of chronic NIV targeted at a substantial arterial carbon dioxide reduction in stable hypercapnic COPD is non-inferior to in-hospital NIV initiation.

## Methods

### Design

In a monocentre, 1:1 randomised controlled parallel-group trial, we tested the hypothesis that home initiation of chronic NIV targeted at a substantial arterial carbon dioxide reduction is non-inferior to in-hospital initiation in COPD patients with CHRF, in terms of daytime arterial carbon dioxide pressure (PaCO_2_) reduction after 6 months. Data monitoring and validation was performed by an independent certified data monitor from the Research Data Support of the University Medical Center Groningen, and a data safety monitoring board was instituted that monitored all adverse events throughout the study.

### Patients

From June 2016 to December 2017, all patients with stable COPD and CHRF referred to or home mechanical ventilation (HMV) centre unit with an indication for chronic NIV were informed about the study. Inclusion criteria were: (1) COPD GOLD (Global Initiative for Chronic Obstructive Lung Disease^19^ stage III or IV (post-bronchodilator Forced Expiratory Volume in 1 second (FEV_1_)/Forced Vital Capacity (FVC)<70% and FEV_1_<50% of predicted); (2) daytime PaCO_2_ at room air>6.0 kilopascal (kPa) in stable condition, defined as no COPD exacerbation during the last 4 weeks, and a pH>7.35; (3) age>18 years; (4) existence of a sufficient social support network making initiation of HMV at home possible and (5) written informed consent. Exclusion criteria were: (1) unstable severe cardiac comorbidities (left ventricular ejection fraction below 45% and unstable angina pectoris complaints); (2) living in a nursing home and (3) previous or current use of Continuous Positive Airway Pressure (CPAP or NIV in the home setting (prior NIV during exacerbations was allowed).

### Outcome parameters

The primary study outcome was the change in PaCO_2_ measured during spontaneous breathing at room air at daytime after 6 months compared with baseline. PaCO_2_ was assessed with arterial blood gas analysis by puncture of the radial artery at least 3 hours after cessation of the nocturnal NIV.

Secondary outcomes were safety, symptoms and health-related quality of life (HRQoL), measured by the Severe Respiratory Insufficiency Questionnaire (SRI),^20^ the Medical Outcomes Study 36-Item Short-Form Health Survey,^21^ the Clinical COPD Questionnaire (CCQ),^22^ the Hospital Anxiety and Depression Scale,^23^ and the Medical Research Council score to assess dyspnoea^24^; lung function^25 26^; exercise tolerance, assessed by the 6 min walking distance^27^; compliance with the ventilator, exacerbation and hospitalisation frequency (assessed by checking medical records from the hospital and the general practitioner and by checking pharmacy read-outs for courses or prednisolone and/or antibiotics prescribed for COPD exacerbations) and costs. The direct (medical and non-medical) and indirect costs of NIV initiation at home were compared with in-hospital NIV initiation over a 6-month period using a societal perspective.^28^ For a detailed explanation of the outcome measures, see online repository.

10.1136/thoraxjnl-2019-213303.supp1 Supplementary data

### Study protocol

#### Initial assessment

All patients visited our outpatient clinic for a baseline assessment of demographics, exercise tolerance, lung function and measurement of daytime gas exchange. All patients underwent an echocardiogram and a polygraphy (at home) prior to NIV initiation to characterise the population (for details see online repository). One of the investigators enrolled patients into the study once they fulfilled all criteria.

#### Hospital NIV initiation

Patients randomised to in-hospital initiation were initiated on chronic NIV according to the regular procedures on our pulmonary ward. We used a bilevel positive airway pressure (BiPAP)-set ventilator (BiPAP A40 and BiPAP A30, Philips Respironics, Murrysville, Pennsylvania, USA) and adjusted the settings to achieve normocapnia during the night or at least a reduction in nocturnal mean PtCO_2_of 20% compared with the first night of spontaneous breathing.^29^ The initiation period was finished and the patient was discharged home once he or she could sleep at least 6 consecutive hours with the ventilator and the gas exchange goals were achieved. When necessary, our specialised nurse joined the patients at home to instal the ventilator.

#### Home NIV initiation

Patients randomised to home initiation were initiated on chronic NIV completely at their home, using telemedicine. Ventilator data were retrieved via a GPRS system clicked on the back of the ventilator (BiPAP A40 and BiPAP A30, Philips Respironics), which sent data to an online platform (Encore Anywhere, Philips Respironics). Changes in ventilator settings could be made remotely. Also, PtCO_2_ was measured (SenTec DM, Software V-STATS V.4.0, SenTec AG, Therwil, Switzerland) and these data were retrieved remotely via a high-end ambulatory remote monitoring device (Dyna-vision, Techmedic International, Broek op Langedijk, the Netherlands). The specialised nurse visited the patient at day 1 to instal the equipment, explain all procedures, and to practice with NIV, and the last day to return the telemedicine/measurement devices and finish the initiation period. For a detailed explanation of the home initiation, see online repository.

#### Follow-up

All patients visited the outpatient clinic 3 months (limited assessment) and 6 months (full assessment) after the NIV was initiated and follow-up measurements were performed by one of the investigators, who were not blinded to the allocation sequence. Patients could contact us by telephone whenever they had any questions.

### Statistics

We defined a non-inferiority margin of 0.4 kPa for the difference in change of our primary endpoint, PaCO2, between home and in-hospital initiation, as a difference in the reduction of less than 0.4 kPa was deemed to be clinically irrelevant to base treatment preference. This decision was made based on previous trials, showing clinically relevant benefits with PaCO_2_ changes of more than 0.45 kPa.^8 12 14 16 17 30 31^ With a one-sided alpha of 0.025, a beta of 0.2, a Standard Deviation (SD) of 0.4^12 16^ and expected drop out rate of 25%,^17^ 62 patients needed to be randomised. Randomisation was performed automatically (ALEA randomisation management, FormsVision BV, Abcoude, the Netherlands) with minimisation for baseline PaCO_2_ (≥7 or <7 kPa) and planned pulmonary rehabilitation (yes/no).

The primary analysis was performed including all patients that had at least a measurement at baseline and after 6 months, irrespective of NIV compliance. Additionally, a per-protocol analysis was performed, including all patients who were compliant with their NIV and who completed the study. Safety analyses were performed on all randomised patients who received NIV.

Differences in baseline variables between the home and in-hospital groups, and between the completers and the drop outs were tested with a t-test or Mann-Whitney U test for continuous variables and χ^2^ tests for categorical variables. To test changes within a group over time, a general linear repeated measures analysis of variance with Bonferroni correction or a paired t-test was performed.

To test the null hypothesis that home initiation is non-inferior to in-hospital initiation, we calculated the absolute change in PaCO_2_ from baseline to 6 months and performed a linear regression analysis with correction for the baseline value calculating the adjusted mean difference between the groups. Other outcomes were tested in the same way. For outcome variables for which the change had a skewed distribution, the difference in change was tested with a Mann-Whitney U test. SPSS V.25.0 was used to perform the analyses.

## Results

### Baseline characteristics

**Figure 1:**
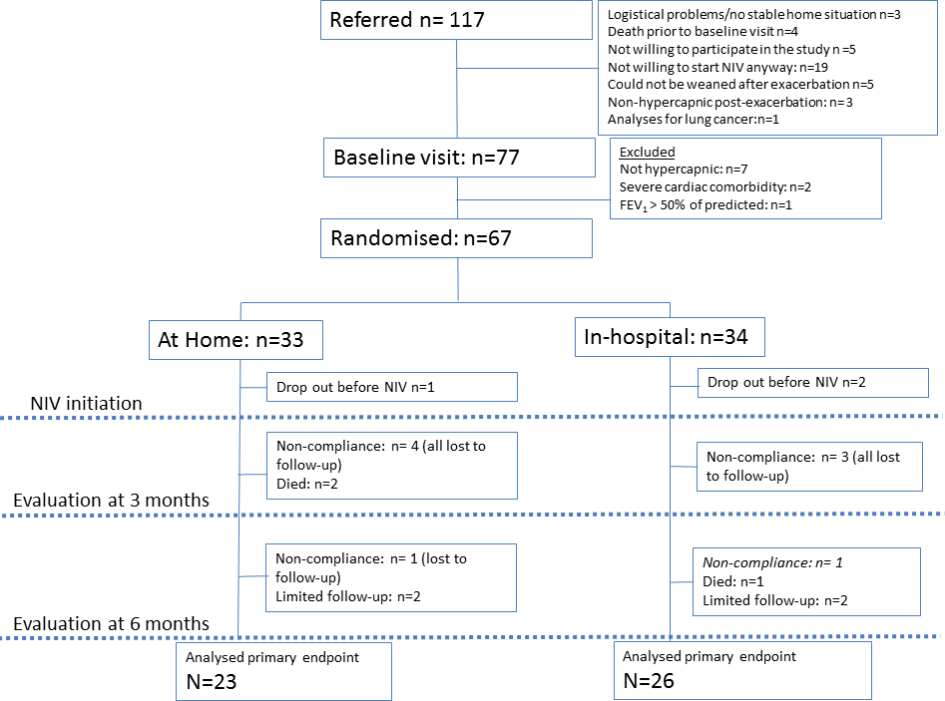
Figure 1Consort diagram of the RECONSIDER trial. One patient that was non-compliant and stopped his NIV was followed for outcome measurements (shown in *italic*). FEV_1_, Forced Expiratory Volume in 1 second; NIV, non-invasive ventilation.

The study flow chart is shown in figure 1. A total of 117 patients were screened as outpatients and 67 were randomised when they met all inclusion criteria, and subsequently planned for NIV initiation as soon as possible. Three patients dropped out during this period before they were initiated on NIV; one patient died, one patient exacerbated and was initiated on NIV acutely and one patient refused to participate further. The ‘waiting period’ was >30 days in 10 out of 31 patients (32%) with a median of 22 days (range 5–62 days) in the in-hospital group, while it was >30 days in 5 out of 30 patients (17%) with a median of 21 days (range 7–53) days in the home group (difference not significant). Five patients in the home group, and four patients in the hospital group, were non-compliant with their NIV. Hospital initiation was offered in the non-compliant home group patients, but none of them wanted an in-hospital attempt. In total, three patients died during NIV therapy. Table 1 presents the baseline characteristics. Comorbidities were prevalent (online supplementary table 1). Patients with unstable cardiac comorbidities or severe heart failure (left ventricular ejection fraction below 45%) were excluded. In the included patients, cardiac function was generally well-preserved and comparable between the home and hospital groups in terms of systolic and diastolic left/right ventricular functional parameters (online supplementary table 1). According to echocardiography guidelines, eight patients in the home group (25%) and seven patients in the hospital group (21%) were considered to have a high probability of pulmonary hypertension (p=0.49; online supplementary table 1).^32^ There were no significant differences between the home and hospital groups (table 1), nor between participants that dropped out and participants that did not drop out, except that the latter group less frequently followed a concurrent pulmonary rehabilitation programme (online supplementary table 2).

**Table 1:** Baseline characteristics of the randomised patients

	Home, N=33	Hospital, N=34	P value
Age, years	63.6±8.6	63.1±7.0	0.80
Male sex, n (%)	15 (45%)	12 (35%)	0.46
Active smokers, n (%)	4 (12%)	9 (27%)	0.22
Packyears	37.4±18.7	47.6±25.8	0.07
LTOT n (%)	26 (79%)	28 (82%)	0.77
BMI, kg/m^2^	24.9±6.0	25.7±4.1	0.52
Inhaled long-acting beta-agonists, n (%)	32 (97%)	31 (91%)	0.61
Inhaled long-acting anti-cholinergics, n (%)	29 (88%)	31 (91%)	0.71
Inhaled corticosteroids, n (%)	27 (82%)	28 (82%)	0.99
Morphine, n (%)	11 (33%)	8 (24%)	0.43
Oral corticosteroids, n (%)	15 (46%)	12 (35%)	0.46
Previous NIV experience during AECOPD, n (%)	16 (48%)	17 (50%)	0.90
Episodes of NIV for AECOPD, median (range)	1 (1–3)	1 (1–2)	0.33
Time span NIV for AECOPD—inclusion, days, median (range)	106 (34–2555)	172 (30–1825)	0.19
Exacerbations, previous 12 months, median (IQR)	4 (1–8)	3 (1–5)	0.31
Hospitalisations, previous 12 months, median (IQR)	1 (0–2)	1 (1–2)	0.86
Rehabilitation, n (%)	7 (21%)	8 (24%)	0.99
ESS score, points	5.8±3.9	7.1±4.6	0.26
AHI (events/hour), median (IQR)	2.9 (0.5–5.6)	1.4 (0.8–2.9)	0.38
Number of patients with AHI>15, n (%)	1 (3%)	2 (6%)	0.99

Data are shown in mean and SD unless otherwise stated.

AECOPD, acute exacerbation of COPD; AHI, apnoea/hypopnea index; BMI, body mass index; ESS, Epworth Sleepiness Scale;IQR, interquartile range; LTOT, long-term oxygen therapy; NIV, non-invasive ventilation.

### Gas exchange

**Figure 2:**
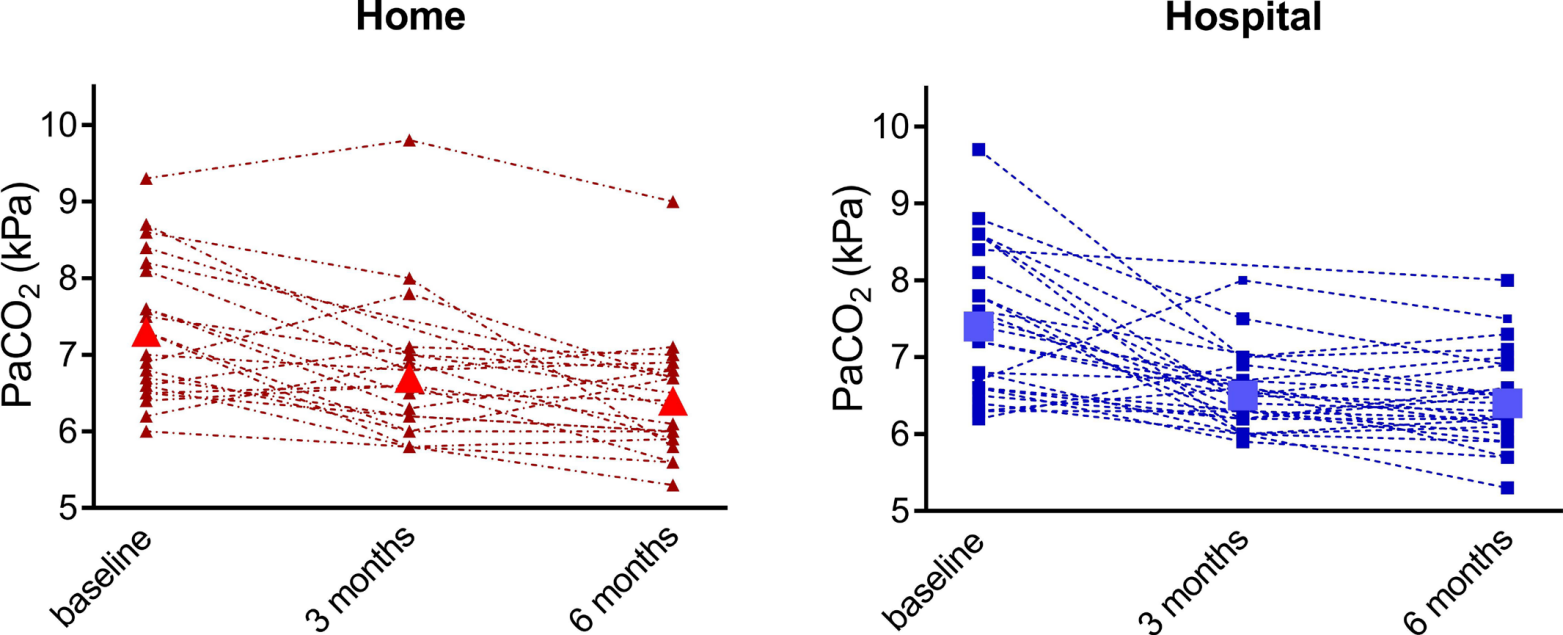
Arterial carbon dioxide pressure (PaCO_2_) at daytime during spontaneous breathing without NIV. Shown are individual patient values of the home and hospital groups and the mean value (home: ∆; hospital: □). 3 mo, 3 months after NIV initiation; 6 mo, 6 months after NIV initiation; NIV, non-invasive ventilation.

In both groups, daytime PaCO_2_ decreased significantly over the 6 months follow-up period (table 2, figure 2). The mean difference in change between the home group versus the hospital group at 6 months was 0.04 kPa (95% CI −0.31 to 0. 38 kPa), showing non-inferiority of home NIV initiation. Other parameters of gas exchange improved in both groups, also without significant differences between groups.

**Table 2:** Gas exchange

	Home, N=23			Hospital, N=26			Adjusted mean difference in change home versus in-hospital (95% CI)
	Baseline	3 months	6 months	Baseline	3 months	6 months	6 months–baseline
PaCO_2_, kPa	7.3±0.9	6.7±0.9*	6.4±0.8**	7.4±1.0	6.5±0.5*	6.4±0.6**	0.04 (−0.31 to 0.38)
PaO_2_, kPa	6.8±1.3	7.5±1.5	7.6±1.2	7.3±1.5	8.1±1.4*	8.0±1.2	−0.18 (−0.85 to 0.49)
HCO_3_^-^, mmol/L	33.1±3.8	30.8±3.2*	29.8±2.9*	33.6±4.2	30.2±2.1*	29.7±2.8**	0.2 (−1.5 to 1.2)

Data are shown as mean±SD. A positive mean difference means an increase from baseline to 6 months for the home compared with the in-hospital group.

Compared with baseline within the group: *p<0.05 and **p<0.001.

HCO_3-_, bicarbonate; kPa, kilopascal; PaCO_2_, partial arterial carbon dioxide pressure; PaO_2_, partial arterial oxygen pressure.

### Lung function

There were no significant differences in improvement in FEV_1_, FVC and lung volumes between the arms (table 3). Both groups improved their 6 min walking distance, but there was no difference between the groups.

**Table 3:** Lung function and exercise tolerance

	Home, N=21		Hospital, N=28		Adjusted mean difference in change home versus in-hospital (95% CI)
	Baseline	6 months	Baseline	6 months	6 months–baseline
FEV_1_, L	0.60±0.16	0.63±0.20	0.60±0.20	0.68±0.30*	−0.05 (−0.14 to 0.03)
FVC, L	2.19±0.55	2.23±0.85	1.94±0.49	2.23±0.90*	−0.31 (−0.67 to 0.05)
TLC, L†	7.4±1.6	7.8±1.5	7.1±1.3	7.3±1.2	0.2 (−0.4 to 0.7)
RV, L	4.9±1.3	5.2±1.2	5.0±1.2	4.9±0.9	0.4 (−0.2 to 0.9)
RV%TLC	65±7	67±10	69±7	67±9*	4 (−0.4 to 8)
6MWD, m	179±93	212±100*	194±85	231±90*	−7 (−49 to 34)

Data are shown as mean±SD. A positive mean difference means an increase from baseline to 6 months for the home compared with the in-hospital group.

*P<0.05.

†Body plethysmography was performed in 16 (home) and 25 (in-hospital) patients, and the 6MWD in 22 (home) and 26 (in-hospital) patients.

FEV_1_, Forced Expiratory Volume in 1 second; FVC, Forced Vital Capacity; 6MWD, 6 min walking distance; RV, residual volume; TLC, total lung capacity.

### Health-related quality of life

**Figure 3:**
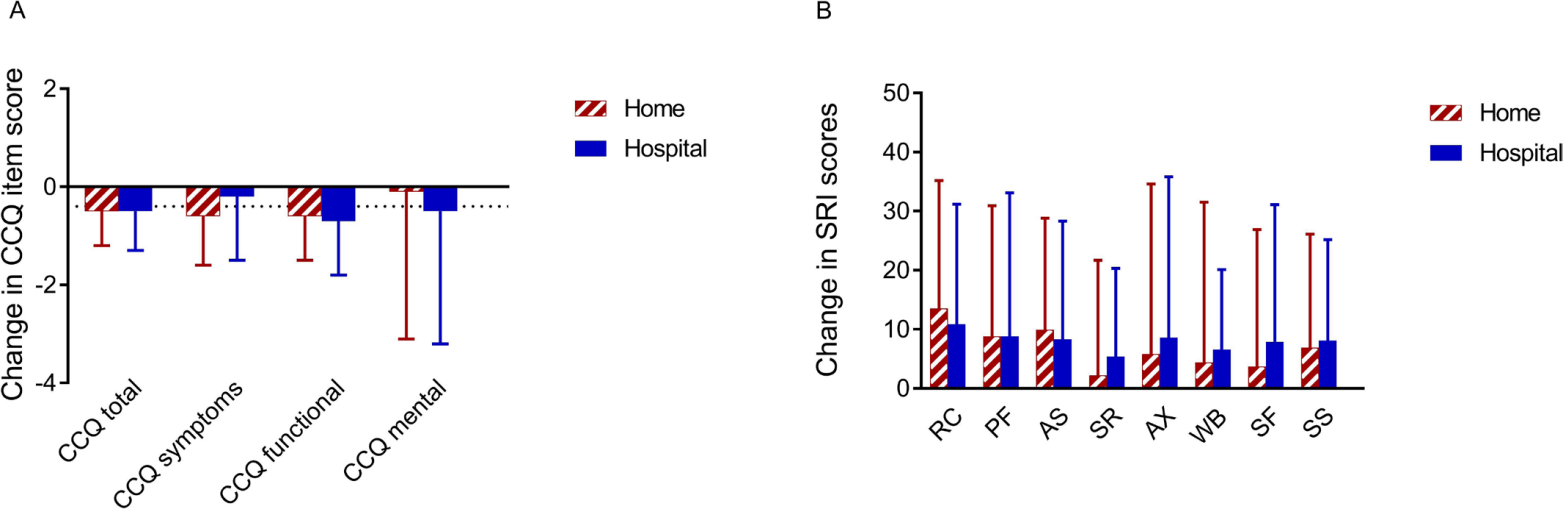
Change in HRQoL per group. Data are shown as mean±SD. For the CCQ, a decrease means an improvement in HRQoL (Minimal Clinical Important Difference −0.4 points). For the SRI, an increase means an improvement in HRQoL. HRQoL: health-related quality of life; CCQ: Clinical COPD Questionnaire; SRI: Severe Respiratory Insufficiency questionnaire, with its domains: RC: respiratory complaints; PF: physical functioning, AS: attendant symptoms and sleep; SR: social relationships; AX: anxiety; WB: psychological well-being; SF: social functioning, SS: summary score.

In both groups, the CCQ improved after 6 months (home group: 3.4±0.8 to 2.9±1.0 points (p<0.05) and hospital group: 3.3±1.0 to 2.8±1.1 points (p<0.05)), without difference between the groups (figure 3). The SRI showed improvements, especially in physical functioning, respiratory complaints and attendant symptoms in both groups (figure 3). The SRI well-being and summary score improved significantly only in the hospital group. Anxiety and depression symptoms and dyspnoea did not change. Nevertheless, for all parameters mentioned, there were no differences in change between the hospital and at-home groups, showing non-inferiority of home initiation with respect to HRQoL and symptoms (online supplementary table 3).

### Exacerbations and hospitalisations for COPD exacerbations

There was no difference in change in number of hospital days, hospitalisation frequency and exacerbation frequency comparing the 6 months prior to inclusion to the study period between the two groups (online supplementary table 4).

### Ventilatory settings, interface, duration of NIV adaptation and compliance

At home, NIV initiation took considerably longer time (14.5 days, range 7–40 days) than in the hospital group (7 days, range 4–15 days; p<0.001). All patients were initiated on a full-face mask, except for three patients in the home group and one patient in the hospital group who were initiated on a nasal mask. Of those patients, only one patient remained on a nasal mask, while the other patients switched during the 6 months follow-up to a full-face mask because of excessive leakage.

In general, patients in the hospital group were ventilated with a higher inspiratory positive airway pressure (IPAP), expiratory positive airway pressure (EPAP) and BURR. In the home group, the IPAP was increased gradually over the follow-up period, while in the hospital group, this was not evident (only a small but significant increase between 3 and 6 months). Therefore, the IPAP–EPAP difference was significantly higher in the hospital group at 3 months, but not at 6 months (table 4).

**Table 4:** Ventilatory settings

Group	Home, N=25			Hospital, N=28		
	Baseline	3 months	6 months	Baseline	3 months	6 months
IPAP, cm H_2_O	21.0±2.8	22.1±2.9*	23.6±2.3*	24.3±3.6†	24.7±3.3†	25.7±3.4*†
EPAP, cm H_2_O	4.5±0.8	4.6±0.9	4.6±0.9	5.7±1.2†	5.8±1.2†	6.0±1.3†
IPAP–EPAP, cm H_2_O	16.5±2.6	17.5±2.5*	19.0±2.1*	18.6±3.3†	18.9±2.8†	19.7±2.7*
BURR, breaths/min	13.5±2.5	13.8±2.2	13.9±2.0	15.6±2.9†	15.3±2.9†	15.4±3.0†

*Significant increase from baseline to 3 months or from 3 months to 6 months.

†Significant difference between the groups at equal time points.

BURR, backup respiratory rate; EPAP, expiratory positive airway pressure;IPAP, inspiratory positive airway pressure.

Compliance with NIV was good. At 3 months, compliance was higher in the home group compared with the hospital group. Patients in the home group used their NIV a mean of 7.7±1.7 hours per day (median: 95% of the total number of days (range 43%–100%)) compared with 6.6±2.1 hours use per day (median: 94% of the total number of days (range 50%–100%)) in the hospital group (p=0.037). Patients initiated in the hospital increased their compliance over time to 7.5±2.0 hours per day (p=0.028) (median percentage of the total number of days 97% (range 81%–100%)) at 6 months, while this remained good in the home group (8.2±1.7 hours per day (median: 99% of the total number of days (range 84%–100%))), so that no significant difference in compliance could be observed at this time point.

### Time investment and costs

**Figure 4:**
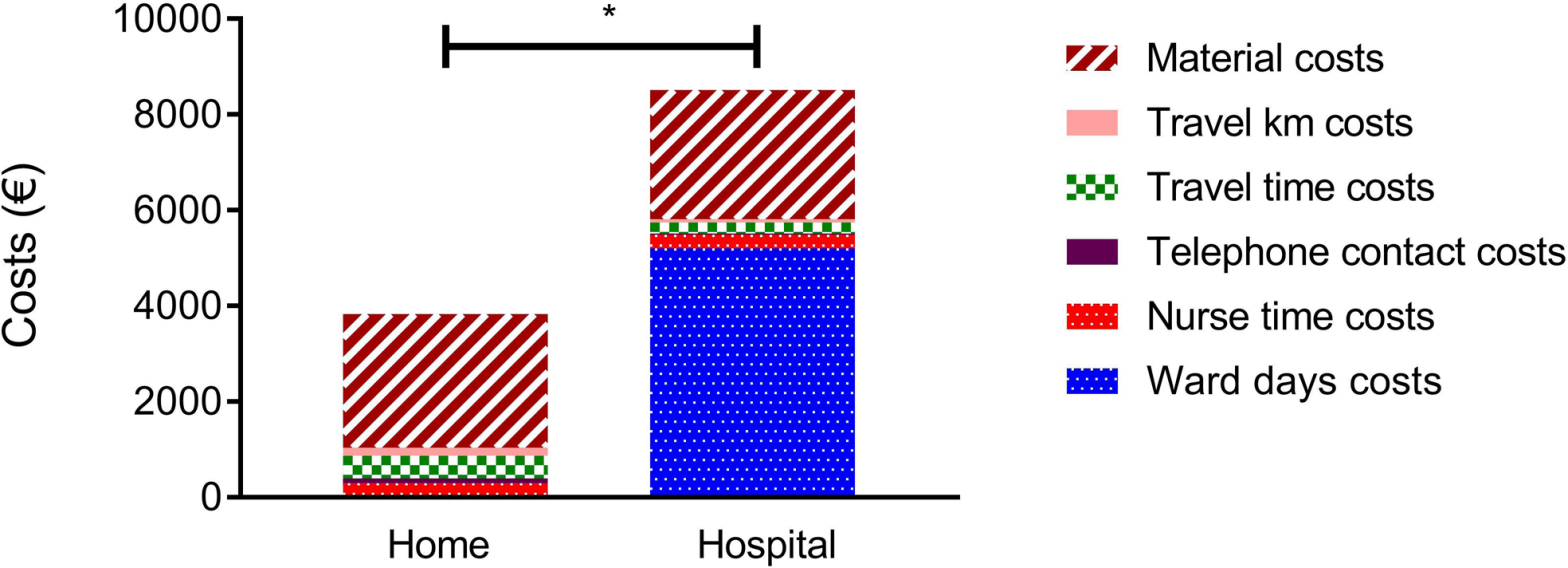
Costs (€) of NIV initiation hospital versus at home. Represented as median costs (€). Material: costs of the ventilator, telemedicine material and material/device for transcutaneous measurements; travel km: costs for travel kilometres of the specialised respiratory nurse; travel time: costs for travel time to the patients of the specialised respiratory nurse; telephone contact: costs for the time spend by the respiratory nurse to have telephone contact with the patients; nurse time: costs of the time spend by the specialised nurse directly with the patient; ward days: costs of the ward days. *p< 0.001. NIV, non-invasive ventilation.

Initiating NIV at home costs less than half (€3768) compared with hospital initiation (€8537), mainly driven by the costs of admission to the respiratory ward (figure 4, table 5). The total time spent directly with the patient was not different between the groups. In the home group, the telephone contact time was longer, as was the number of kilometres driven and the travel time of the nurse to travel to patients’ homes. Differences occurred only during the initiation period, as costs during the follow-up were not different between groups.

**Table 5:** Total costs (€) per patient from NIV initiation to 6 months follow-up

Group	Home, N=25		Hospital, N=28	
Devices	Units	Cost, €	Units	Cost, €
BiPAP A30	1	2500	1	2500
GPRS unit/Telemonitoring*	–	19.2	–	–
Dyna-vision†	–	78	–	–
Initiation period	Units	Cost, €	Units	Cost, €
Ward days for NIV initiation, n	–	–	7.5 (4–15)	4815 (2568–9630)¶
Nocturnal PtCO_2_ measurement‡, n/patient	3 (2.5–4)	108 (90–144)	3 (3–4)	108 (108–144)
Ventilatory specialist time directly with the patient, min	250 (205–323)	187 (153–241)	275 (230–314)	206 (172–235)
Telephone contact time (including calls directly after discharge), min	85 (68–108)	64 (50–80)	0 (0–10)¶	0 (0–7)¶
Travel time by nurse, min	405 (262–482)	303 (196–361)	0 (0–200)¶	0 (0–150)¶
Travel km by nurse	620 (278–722)	118 (53–137)	0 (0–60)¶	0 (0–11)¶
Travel km by patient	–	–	215 (24–327)¶	41 (5–62)¶
Follow-up period	Units	Cost, €	Units	Cost, €
Nocturnal PtCO_2_ measurement‡, n/patient	2 (2–3)	72 (72–108)	2 (2–2.75)	72 (72–99)
Ventilatory specialist time directly with the patient, min	80 (35–168)	60 (26–125)	105 (45–146)	79 (34–109)
Telephone contact time, min	30 (10–65)	22 (0–135)	20 (0–44)	15 (0–82)
Travel time by nurse, min	160 (85–364)	120 (64–272)	195 (54–355)	146 (40–266)
Travel km by nurse	237 (36–600)	45 (7–114)	219 (36–496)	42 (7–94)
Travel km by patient	320 (237–675)	61 (45–128)	270 (139–552)	51 (26–105)
Work productivity§	–	–	–	–
Total medical costs		3709 (3480–4029)		8406 (7490–9003)
Total costs (medical and non-medical)		3768 (3546–4163)		8537 (7540–9175)

Data are presented as median (IQR) as the distribution of the data was skewed.

*The selling price of the GPRS unit was €480, depreciation of €48 per year; we used 5 units for 25 patients to be initiated in 2 years=€480, that is, in total=€19.20/patient. Running of the GPRS unit was included in this price.

†The Dyna-vision selling price is ~€1000, depreciation of €100 per year; we used 2 devices for 2 years to initiate 25 patients=€400=€16 euro per patient. The Dyna-vision requires a service for the online platform which costs €62/months=€62*25 months=€1550 in total=€62/patient.

‡The transcutaneous monitor selling price is ~€10 868, depreciation of €2604 per year. As these devices are used very frequently in regular care, we estimated that depreciation per night use was €11 (estimated use: 240 nights per year). Additionally, costs for material (sensor/membrane, etc) were estimated at €25, resulting in €36 per measurement.

§Only one patient still had a paid job and therefore, these costs were not taken into account.

¶ P <0.05

BiPAP, bilevel positive airway pressure; NIV, non-invasive ventilation; PtCO_2_, transcutaneous carbon dioxide.

### Safety analysis and technical problems

In the hospital group, one patient (3%) died at home unexpectedly after 199 days of successful NIV. In the home group, two patients (6%) died; one patient died 3 days after formal NIV initiation suddenly at home because of respiratory failure (she used her NIV only once at daytime for 10 min and only at day 1) and one patient decided for euthanasia after 20 days NIV at home, while having improved gas exchange but without subjective benefit.

At home, technical problems with nocturnal measurements and transfer of transcutaneous gas exchange data occurred frequently, necessitating additional home visits or daytime measurements. Nocturnal hypoventilation was corrected equally between the groups (online supplementary table 5).

Side effects of NIV occurred in seven (28%) and five (18%) of the patients in the home and hospital groups (non-significant), respectively, and consisted of aerophagia (two vs two patients), mask decubitus (three vs three patients), excessive dyspnoea when NIV was disconnected in the morning (so-called ‘deventilation’ dyspnoea (one patient)) and a dry mouth (one patient). Side effects were a reason to discontinue NIV (intermittently) in two patients, while in the other patients, the problems were either accepted or solved by changing the settings or the mask.

### Per-protocol analysis of the primary outcome

Per-protocol analysis, excluding all non-compliant patients, showed similar results (online supplementary table 6).

## Discussion

This is the first study showing that home initiation of chronic NIV in stable hypercapnic COPD patients, with the use of telemedicine, is non-inferior to in-hospital initiation, safe and reduces costs by over 50%.

In most countries, NIV is initiated in the hospital,^33^ especially in COPD patients who require high-intensity NIV. It is generally thought that NIV initiation should be hospital-based, but there is little consensus on how and where it should exactly be organised: the settings in which it is done (ie, pulmonary ward, respiratory care unit and intensive care unit) vary considerably, as do the costs. We hypothesised that NIV initiation in COPD requires careful titration and monitoring, but not necessarily in the hospital. A recent trial showed that nurse-led overnight NIV titration using transcutaneous oxi-capnometry was even more effective than extensive polysomnographic monitoring and retrospective NIV modification,^34^ as is sometimes thought to be necessary. We also monitored only gas exchange and ventilator data, although retrospectively on a daily basis, as we did not experience the time-pressure of early hospital discharge. We showed that this way of telemedicine-based monitoring is feasible at home. At home, patients are allowed to take more time, resulting in a more relaxed way of getting used to the high pressures necessary to reach the targets, that is, improvement in gas exchange, respiratory muscle unloading, comfort and patient compliance.

NIV initiation at home has been investigated before,^18 35–37^ but not in COPD. Previously, we showed that initiation of NIV can safely be performed at home in patients with neuromuscular and restrictive thoracic diseases. Initiation of NIV at home was preferred by the patients, was equally effective and saved costs compared with inpatient initiation.^18^ In line with these findings, all patients recruited in the present study were, of course, willing to accept the possibility of home initiation; in fact, in 64 of 67 included patients, this was the option they hoped for.

We observed that ventilatory pressures and BURR were higher in the hospital group. Overall, this might be an explanation for the tendency to more improvement in lung function in the hospital group compared with the home group. Of note, in one-third of the transcutaneous gas exchange measurements, a technical problem hindered either reliable measurement of PtCO_2_ at home or the transfer of the data to the hospital. This resulted in extra home visits (and extra costs) and, although much effort was done to prevent this, probably less strict CO_2_-directed NIV titration initially. Although this surely needs to be improved, after 6 months, there was no difference between home and hospital initiation in change in our primary outcome, that is, daytime PaCO_2_, nor in HRQoL or exercise tolerance. We hypothesise that the slightly lower pressures might have been counterbalanced by the slightly better compliance in the home group. Overall, differences in clinical outcomes from a clinical point of view were small, and, in our opinion, were more than balanced by the financially and patient-preferred approach of at-home NIV initiation.

Drop out rates tended to be higher in the home group compared with the hospital group. However, drop out rates (22% in the home group and 16% in the hospital group) from the included severe COPD population were comparable to drop out rates in regular care in our centre (overall~15%–25% within 6 months). Of note, but not surprisingly, adding a structured pulmonary rehabilitation programme after NIV initiation, as promoted in our centre, seemed to be of benefit to increase compliance. There was no difference in deaths, two versus one patient; the total number being in line with the severity of their baseline chronic respiratory failure and severely reduced lung function.^12^ Exacerbation frequency did not change, although there seemed to be a reduction in hospitalisation days after NIV initiation at home. However, the study was neither designed nor powered to find an effect on exacerbations/hospitalisations, and therefore, these results should be considered cautiously.

Limitations of the present study are that it was a monocentre study in a centre that has a large experience with home initiation of NIV; that home initiation was performed by three experienced ventilatory nurse specialists and that the telemedicine set up used to initiate patients at home included a strict monitoring protocol. Therefore, we can only conclude that home initiation including this intensive monitoring set up was non-inferior to in-hospital initiation. Second, study results cannot be generalised to patients who need NIV directly after a COPD exacerbation, as all included patients were initiated at least 4 weeks after an exacerbation. Nevertheless, the study is representative of the severe COPD population; we included patients with severe hypercapnia, and although we did not include ‘post-exacerbation’ patients, we included both patients who frequently exacerbated before, also needing acute NIV, and patients with less frequent prior exacerbations not having prior NIV experience. In both groups, home initiation was feasible. As the number of patients with COPD/obstructive sleep apnea overlap syndrome was very small, the study results cannot be formally generalised to these patients. Nevertheless, NIV set up did work out very well in our overlap patients. The costs calculations depend on the local-specific and country-specific situation, such as the actual setting of NIV initiation (pulmonary ward, medium care unit and intensive care unit), duration of stay at the ward, health insurance fees for outpatient care and the distance that caregivers have to travel. However, even if the number of ward days would be 2 days (one night), the costs would still be higher with hospital initiation. Moving the majority of NIV initiations from hospital to home will necessitate a change in the staffing model. Most importantly, patient preference with home initiation might be an at least equally important outcome that results in benefit even with equal costs.

To conclude, we showed for the first time that home initiation of NIV with the use of telemedicine in COPD patients with CHRF is non-inferior to hospital initiation, safe and associated with savings of over 50% of the costs.
