# Supplementary material for: Home initiation of chronic non-invasive ventilation in COPD patients with chronic hypercapnic respiratory failure: a randomised controlled trial
Source: Thorax. 2019 Sep 4;75(3):244–52. doi: 10.1136/thoraxjnl-2019-213303 (PMC7063397; doi:10.1136/thoraxjnl-2019-213303)
Supplement: Supplementary data [file thoraxjnl-2019-213303supp001.pdf]

**ONLINE SUPPLEMENTARY REPOSITORY**

# Home initiation of chronic noninvasive ventilation in COPD patients with chronic hypercapnic respiratory failure: a randomised controlled trial

Marieke L. Duiverman,[1,2] Judith M. Vonk,[2,3] Gerrie Bladder,[1,2] Joost P. van Melle,[4] Jellie Nieuwenhuis,[1,2] Anda Hazenberg,[1,2] Huib A.M. Kerstjens,[1,2] Job F.M. van Boven,[2,4] , Peter J.Wijkstra[1,2]

[1]Department of Pulmonary Diseases/Home Mechanical Ventilation, University of Groningen, University Medical Center Groningen, Groningen, the Netherlands

[2]Groningen Research Institute for Asthma and COPD (GRIAC), University of Groningen, University Medical Center Groningen, Groningen, the Netherlands

[3] Department of Epidemiology, University of Groningen, University Medical Center Groningen, Groningen, the Netherlands

[4] Thorax center, Department of Cardiology, University of Groningen, University Medical Center Groningen, Groningen, the Netherlands

[5] Department of General Practice & Elderly Care Medicine, University of Groningen, University Medical Center Groningen, Groningen, the Netherlands

Word count online supplement: 1937

# METHODS

## Patients

Patients with COPD GOLD (Global initiative for Chronic Obstructive Lung Disease) stage III or IV (post-bronchodilator Forced Expiratory Volume in 1 second ($FEV_1$)/ Forced Vital Capacity (FVC) < 70% and $FEV_1$< 50% of predicted) and daytime $PaCO_2$ at room air > 6.0 kiloPascal (kPa) in stable condition, defined as no COPD exacerbation during the last 4 weeks, and a pH > 7.35; were included. In the Netherlands, current indications for chronic NIV in COPD are 1) chronic hypercapnic respiratory failure ($PaCO_2$ > 6.0 kPa); as RCTs have shown positive results in this group, also in the patients with more mild hypercapnia; and 2) persistent severe hypercapnia after a COPD exacerbation, according to the trial of Murphy et al, which is fortunately a minority of the referred patients. For the present study was set up to fulfil the needs of patients in a stable condition.

## Measurements

### Assessment of comorbidities

Comorbidities were assessed by a structured patient interview, revealing patient reported comorbidities, combined with a review of medication specific use. The following comorbidities were assessed: osteoporosis (non-traumatic fractures and/or bisphosphonate use), cardiac comorbidities, urogenital problems, diabetes, anxiety (symptoms and/or benzodiazepines use) and depression (symptoms and/or anti-depressants use), sleep apnoea.

*Cardiac comorbidity.*

In all patients, an echocardiogram was performed based on a pre-specified protocol using a Vivid E9 (GE, Horton, Norway) and a 2.5-3.5 MHz probe. Images of at least 3 cardiac cycles were digitally stored in a raw DICOM format for offline analysis. Offline analysis was performed on a EchoPac workstation (GE, BT12). Evaluation of cardiac structure included cardiac dimensions and wall thickness as well as left ventricular and left atrial volumes. Cardiac function analysis consisted of conventional volumetric measurements (left



Duiverman ML, *et al. Thorax* 2020; 75:244–252. doi: 10.1136/thoraxjnl-2019-213303

ventricular ejection fraction (LVEF)) for LV systolic function, mitral valve inflow parameters and pulsed wave tissue Doppler early velocity for the evaluation of LV diastolic function. Right ventricular dimension and function were evaluated by end-diastolic diameter and tricuspid annular plane systolic excursion (TAPSE) and RV systolic tissue velocity (RV s'). Measurements were performed in accordance with the current recommendations and guidelines for the evaluation of cardiac structure and function.[1, 2]

**Measurement of health-related quality of life (HRQoL) and symptoms**

Both disease specific and generic HRQoL was assessed.

Generic HRQoL was measured with the Medical Outcomes Study 36-Item Short-Form Health Survey, the SF-36. The SF-36 consists of 36 items divided into eight domains: physical functioning, role-physical, bodily pain, general health, vitality, social functioning, role-emotional, and mental health. For each domain, scores range from 0 (worst) to 100 points (best).[3] These summary measure scores are transformed to create a minimum and maximum possible score of 0 and 100 points. All scores below 50 points can be interpreted as below the general population norm.[3]

Disease specific HRQoL was assessed by the Severe Respiratory Insufficientie questionnaire (SRI) and the Clinical COPD Questionnaire (CCQ). The SRI is a 49-item questionnaire specifically designed for patients with CHRF measuring 7 conceptual domains of HRQoL: respiratory complaints, physical functioning, attendant symptoms and sleep, social relationships, anxiety, psychological well-being and social functioning, and a summary score. The domain scores are calculated by transforming the mean item score into a percentage ranging from 0 (most severely depressed health status as complaints/limitations are severe) to 100 (perfect health status with no complaints/limitations).[4]

The CCQ is a disease specific but not "respiratory failure" specific questionnaire. The CCQ is a 10-item, self-administered questionnaire that can be completed in less than 2 minutes. Items are divided into three domains: symptom, functional state and mental state; patients are required to respond to each item on a seven-point Likert scale where 0 = asymptomatic/no limitation and 6 = extremely symptomatic/total limitation. The final score is the mean of all ten items, and scores for the three domains can be calculated separately if required.[5]



Duiverman ML, *et al. Thorax* 2020; 75:244–252. doi: 10.1136/thoraxjnl-2019-213303

Depression and anxiety symptoms were assessed with the Hospital Anxiety and Depression Scale (HADS).[6-8] The HADS is composed of two 7-item subscales (HADS-D and HADS-A for depression and anxiety, resp.) both ranging from 0 to 21 with higher scores indicating more severe distress. Items enquire about symptoms over the preceding week and are self- or clinician-rated on a 4-point Likert scale. The optimal cut-off score was considered 8 points both for the anxiety and depression domain.

Dyspnoea was assessed by the MRC score, ranging from 0 (no dyspnoea) to 5 (dyspnoea at rest).[9]

Lung Function

Lung function was assessed according to international guidelines by spirometry (forced expiratory volume in 1 second ($FEV_1$), forced vital capacity (FVC)),[10] bodyplethysmography (total lung capacity (TLC), residual volume (RV)),[11] and respiratory muscle testing (maximal inspiratory pressure ($P_I max$)).[12]

Exercise tolerance

Exercise tolerance was assessed with the 6-minute walking test, performed according to the European Respiratory Society/American Thoracic Society guidelines,[13] along a 30 m indoor course, with standardised encouragements given by the investigator. Oxygen was prescribed in the regular amount advised to the patient during exercise and remained stable over the 2 different time points. Oxygen saturation, heart rate, and Borg dyspnoea scores[14] were assessed prior to the test and at the end of the test.

Calculation of costs

Costs were separately calculated for the direct medical costs and the nonmedical costs. In line with Dutch health economic guideline recommendations, standard medical costing tariffs (from the Costing Manual)[16] were applied, which reflect nationally representative cost prices for actual daily medical care.

Direct medical costs included:

1) healthcare costs for NIV-related medical equipment, such as the costs for the ventilatory device, costs for the transcutaneous measurements and costs for telemonitoring equipment (the latter only in the home group). The same ventilator was used in both



Duiverman ML, et al. Thorax 2020; 75:244–252. doi: 10.1136/thoraxjnl-2019-213303

groups (BiPAP A40® and BiPAP A30®, Philips Respironics, Murrysville, PA, USA). As almost all patient used a full-face mask, we did not take these costs in account. In the hospital-group, we registered the of amount days admitted to the hospital ward in the academic hospital.

2) In both groups, the following direct non-medical parameters were registered:
   a) the caregiver time spent with the patient to initiate, titrate and adapt the NIV during the set-up and during the follow-up. Also in hospital, this is performed by a specialised nurse from our HMV department and as so, is not included in the standard ward day tariff.
   b) the amount of km driven by the caregiver to the homes of the patients to initiate them (in the home group) and to follow them up (in both groups). In some patients in the hospital group, the specialized nurse placed the ventilator at home on the day of discharge.
   c) the travel time for the caregivers to do this.
   d) the telephone contact time in both groups during initiation and follow-up.

Indirect costs included the amount of km driven by the patient to visit the hospital (only in the inpatient group). The amount of work absence by the patients was not taken into account as only one 1 patient still had a paid job. Also, intercurrent exacerbations were not taken into account as these did not differ between the groups.

Other indirect (medical) costs (e.g. medical costs in life-years gained) were not considered in the current analysis, as mortality rates were not different during study follow-up. All healthcare utilization was multiplied by the standard unit prices of care from the Dutch manual for costing research. If unit prices were not documented in the guidelines, we estimated them according to the methods stated in the guidelines.[15, 16] For each kilometre driven, € 0.19 was calculated (both for the nurse and the patients). The hourly wage of the nurse practitioner was calculated from the net hourly salary for a specialise nurse in the Netherlands and was calculated to be €32.30 per hour. To adjust for extra costs for the employer (i.e. insurance premiums, retirement funds etc.), the guidelines advice a correction factor of 39%, making the total hourly wage become: 32.30 + (0.39 * 32.30) = €44.90 per hour. Valuation of costs being admitted to the ward per day in an academic hospital in the

Netherlands was, according to the guidelines,[16] estimated to be €642 per day (Zorginstituut Nederland (Healthcare Institute the Netherlands) (ZiN)) 2014 reference price).

**Home initiation of NIV**

Patients randomised to home initiation were initiated completely at their home. In order to make this possible we used telemedicine. Ventilator data could be retrieved via a GPRS system clicked on the back of the ventilator (A30® or A40®, Philips, the Netherlands), that sent data to an online platform (Encore Anywhere®, Philips, the Netherlands). Changes in ventilator settings could be made remotely. Also, transcutaneous gas exchange was measured (SenTec DM®, Software V-STATS 4.0; SenTec AG; Therwil, Switzerland) and these data were retrieved remotely via a high-end ambulatory remote monitoring device (Dyna-vision®, Techmedic international, Broek op Langedijk, the Netherlands).

To standardize implementation and patient education procedures, 3 ventilatory specialist nurses initiated all patients at home. They were replaced in clinical care, so that standard care (in-hospital initiation) was not differently staffed compared to prior to the study start. The maximum set-up numbers were limited to 2 per week, but mainly because of the availability of telemonitoring equipment.

At day 1, the ventilatory specialist nurses brought the equipment, explained all procedures, practiced and titrated patients at daytime and consented with the patients on a practice scheme. Similar to the inpatient patients, transcutaneous $CO_2$ was measured the first night during spontaneous breathing. At day 2, patients started to practice and started to use the NIV at night. The nurse called the patients every morning except for weekend days to discuss how they were doing and to discuss adjustments that he/she wanted to make according to the daily read-outs of ventilator settings and compliance. Once patients could sleep at least 6 hours with the ventilator, they were instructed to connect the transcutaneous monitor again, and the nurse reviewed these data also the next morning. According to the results, further adjustments were made remotely, and another transcutaneous measurement was performed. On weekend and national celebration days, patients were not contacted nor were any measurements done; patients were instructed the day before to keep practicing and trying to increase the number of hours on NIV. Once the goals, set exactly the same as in



Duiverman ML, *et al. Thorax* 2020; 75:244–252. doi: 10.1136/thoraxjnl-2019-213303

the inpatient group, were achieved the nurse visited the patient again to finish the initiation period and to collect the GPRS add-on system, the transcutaneous monitor and Dyna-vision. If patients were admitted for pulmonary rehabilitation afterwards, no further changes in settings were made.



Duiverman ML, *et al. Thorax* 2020; 75:244–252. doi: 10.1136/thoraxjnl-2019-213303

# RESULTS

**Table 1-online:** Comorbidities of the randomised patients

| | Home N=33 | Hospital N=34 | P-value |
|---|---|---|---|
| Osteoporosis, n (%) | 12 (36%) | 12 (35%) | 0.85 |
| Atherosclerosis, n (%) | 15 (45%) | 13 (38%) | 0.55 |
| Diabetes Mellitus, n (%) | 7 (21%) | 5 (15%) | 0.45 |
| Depression, n (%) | 15 (45%) | 12 (35%) | 0.40 |
| Anxiety, n (%) | 18 (54%) | 15 (44%) | 0.39 |
| Arterial hypertension, n (%) | 20 (60%) | 20 (59%) | 0.88 |
| Echocardiography | | | |
| Left ventricle and atrium | | | |
| Left ventricular ejection fraction*, % | 59 ± 4 | 58 ± 5 | 0.32 |
| LAVi (ml/m2) | 27.3 ± 11 | 25.1 ± 8 | 0.42 |
| LVMi (g/m2) | 71.5 ± 30 | 73.9 ± 27 | 0.98 |
| $E/e'_{mean}$ | 9.0 ± 3.1 | 8.2 ± 2.0 | 0.11 |
| $e'_{,\ lateral}$ | 9.2 ± 2.5 | 10.1 4.1 | 0.59 |
| $e'_{,\ septal}$ | 8.7 ± 2.9 | 7.7 ± 1.7 | 0.23 |
| Right ventricle | | | |
| TAPSE (mm)# | 21.0 ± 3.8 | 19.6 ± 4.6 | 0.22 |
| RV s' (cm/s) ## | 12.7 ± 3.4 | 11.6 ± 2.6 | 0.19 |
| TR V max (m/s) ###, median (range) | 3.2 (2.1-3.9) | 3.4 (2.5-4.4) | 0.31 |
| sPAP### (mmHg), median (range) | 48 (23-67) | 47 (26-80) | 0.62 |
| NTproBNP, ng/L, median (IQR) | 110 (59-190) | 116 (70-288) | 0.37 |

**Legend table 1:** data are shown in mean and standard deviation (SD) unless otherwise stated. Left ventricular ejection fraction: *: reliable measurements were obtained in 25 (home) vs. 26 (hospital patients; LAVi: left atrial volume index (increased: > 34 ml/m$^2$), LVMi: left ventricular mass index (increased: ≥ 115 g/m$^2$ for males and ≥ 95 g/m$^2$ for females), $E/e'_{mean}$: ratio of early diastolic mitral valve inflow to early diastolic tissue velocity



Duiverman ML, *et al. Thorax* 2020; 75:244–252. doi: 10.1136/thoraxjnl-2019-213303

(increased $E/e'_{mean}$: ≥13); e': early diastolic tissue velocity (decreased e': <9 cm/s)[17]; #: TAPSE: tricuspid annular plane systolic excursion (could be measured reliably in 31 (home) and 30 (hospital) patients); ## :RV s': right ventricular systolic tissue velocity (could be measured reliably in 24 (home) and 26 (hospital) patients); ### :TR Vmax: maximum tricuspid regurgitation velocity; sPAP: systolic pulmonary artery pressure (was measured in 8 and 11 patients in the home and hospital group as TR Vmax was none in 15 patients (47%) in the home group and 13 (39%) patients in the hospital group and could not be measured reliably in 7 and 12 patients); NTproBNP: N-terminal of brain natriuretic peptide.



Duiverman ML, *et al. Thorax* 2020; 75:244–252. doi: 10.1136/thoraxjnl-2019-213303

**Table 2-online:** Baseline characteristics of the patients that dropped out versus the patient that completed the study

| | **Completed the study N=52** | **Dropped-out N=15** | **P-value Drop-out vs. completers** |
|---|---|---|---|
| NIV initiation at home, n (%) | 25 (48%) | 8 (53%) | 0.776 |
| Age, years | 62.8 ± 7.0 | 65.3 ± 10.1 | 0.278 |
| Male sex, n (%) | 21 (40) | 6 (40) | 0.611 |
| Active smokers, n (%) | 9 (17) | 4 (27) | 0.319 |
| Packyears, n | 41.6 ± 23.6 | 46.0 ± 21.2 | 0.510 |
| LTOT (%) | 44 (85) | 10 (67) | 0.146 |
| BMI, kg/m2 | 25.7 ± 4.9 | 23.8 ± 5.6 | 0.201 |
| Inhaled long-acting beta-agonists, n (%) | 49 (94) | 14 (93) | 0.647 |
| Inhaled long-acting anti-cholinergics, n (%) | 46 (88) | 14 (93) | 0.505 |
| Inhaled corticosteroids, n (%) | 43 (83) | 12 (80) | 0.538 |
| Morphine, n (%) | 13 (25) | 6 (40) | 0.332 |
| Oral corticosteroids, n (%) | 18 (35) | 6 (40) | 0.133 |
| Rehabilitation, n (%) | 15 (29) | 0 (0) | **0.013** |
| ESS score, points | 6.6 ± 4.4 | 6.0 ± 4.4 | 0.702 |
| AHI, events/h, median (IQR) | 1.9 (1-5) | 1.2 (0-7) | 0.568 |
| Number of patients with AHI > 15, n (%) | 3 (6) | 0 (0) | 0.470 |
| $PaCO_2$ baseline, kPa | 7.4 ± 0.9 | 7.4 ± 1.4 | 0.964 |
| $PaO_2$ baseline, kPa | 7.1 ± 1.4 | 6.8 ± 2.0 | 0.625 |
| $HCO_3$ baseline, kPa | 33.4 ± 3.8 | 35.1 ± 9.4 | 0.305 |
| FEV1 baseline, kPa | 0.59 ± 0.18 | 0.60 ± 0.20 | 0.954 |
| FVC baseline, kPa | 2.05 ± 0.54 | 2.06 ± 0.86 | 0.961 |
| RV%TLC, baseline | 68 ± 7 | 68 ± 7 | 0.900 |



Duiverman ML, *et al. Thorax* 2020; 75:244–252. doi: 10.1136/thoraxjnl-2019-213303

| | | | |
|---|---|---|---|
| 6MWD, baseline, meter | 181 ± 90 | 174 ± 113 | 0.794 |
| CCQ total score | 3.4 ± 1.0 | 3.4 ± 0.9 | 0.918 |
| SRI summary score | 48 ± 14 | 46 ± 10 | 0.605 |
| HADS total score | 15.4 ± 9 | 16.2 ± 5 | 0. 758 |

**Legend table 2:** data are shown in mean and standard deviation (SD) unless otherwise stated. BMI: body mass index; LTOT: long-term oxygen therapy; ESS: Epworth sleepiness scale; AHI: apnoea/hypopnea index; $PaCO_2$; partial arterial carbon dioxide pressure; $PaO_2$: partial arterial oxygen pressure; $HCO_3$: bicarbonate; $FEV_1$: forced expiratory volume in 1 second; FVC: forced vital capacity; TLC: total lung capacity; RV: residual volume; 6MWD: 6 minute walking distance; CCQ: clinical COPD Questionnaire; SRI: Severe Respiratory Insufficiency questionnaire; HADS: hospital anxiety and depression scale.

**Table 3-online: Symptoms and HRQoL**

| | | Home N=25 | | | Hospital N=28 | | | Adjusted mean difference in change Home vs. in-hospital (95% CI) |
|---|---|---|---|---|---|---|---|---|
| | | **Baseline** | **6 months** | **Change 6 months-baseline** | **Baseline** | **6 months** | **Change 6 months-baseline** | |
| **HADS** | **Total** | 16.0 ± 7.1 | 15.2 ± 6.6 | -0.8 ± 8.3 | 15.1± 9.9 | 13.0 ± 8.7 | -2.2 ± 7.3 | -1.8 (-6 to 2) |
| | **ANX** | 8.2 ± 4.9 | 7.9 ± 3.4 | -0.3 ± 4.9 | 6.9 ± 5.8 | 5.9 ± 4.8 | -1.0 ± 4.1 | -1.4 (-3 to 1) |
| | **DEPR** | 7.9 ± 3.5 | 7.4 ± 3.7 | -0.5 ± 4.7 | 8.3 ± 4.6 | 7.2 ± 4.3 | -1.1 ± 3.9 | -0.4 (-3 to 2) |
| **CCQ** | **Symp** | 3.2 ± 1.0 | 2.6 ± 1.1* | -0.6 ± 1.0 | 3.1 ± 1.2 | 2.9 ± 1.1 | -0.2 ± 1.3 | 0.4 (-0.3 to 0.9) |
| | **Func** | 4.2 ± 1.1 | 3.6 ± 1.1* | -0.6 ± 0.9 | 4.1 ± 1.2 | 3.4 ± 1.4* | -0.7 ± 1.1 | -0.2 (-0.8 to 0.4) |
| | **Mental** | 2.3 ± 1.3 | 2.1 ± 1.5 | -0.1 ± 3.0 | 2.1 ± 1.9 | 1.5 ± 1.6 | -0.5 ± 2.7 | -0.7 (-2.3 to 1.0) |
| | **Total** | 3.4 ± 0.8 | 2.9 ±1.0* | -0.5 ± 0.7 | 3.3 ± 1.1 | 2.8 ± 1.1* | -0.5 ± 0.8 | 0.0 (-0.4 to 0.5) |
| **MRC** | | 5 (3.5-5) | 5 (4-5) | 0.1 ± 1.2 | 4 (4-5) | 5 (4-5) | 0.0 ± 1.3 | 0.2 (-0.4 to 0.7) |
| **SF36#** | **PF** | 10 (5-25) | 20 (5-35) | 9 ± 14 | 8 (0-15) | 12.5 (0-30) | 9 ± 19 | 0.0 (-10 to 11) |

| | | | | | | | | |
|---|---|---|---|---|---|---|---|---|
| | **RP** | 0 (0-0) | 0 (0-25) | 11 ± 25 | 0 (0-6) | 0 (0-25) | 8 ± 25 | 0.8 (-15 to 17) |
| | **RE** | 33.3 (0-100) | 16.7 (0-100) | - 2 ± 55 | 0 (0-100) | 50 (0-100) | 17 ± 45 | -13.5 (-39 to 12) |
| | **BP** | 72 (26-84) | 41 (32-82) | - 5 ± 25 | 74 (31-100) | 67 (39-81) | -2 ± 28 | -5.3 (-19 to 9) |
| | **MH** | 68 (48-72) | 60 (52-76) | - 3 ± 20 | 64 (59-81) | 70 (55-89) | 4 ± 15 | -7.9 (-17 to 2) |
| | **V** | 72 (26-84) | 41 (32-82) | 7 ± 15 | 74 (31-100) | 67 (39-81) | 4 ± 18 | 2.6 (-7 to 12) |
| | **SF** | 50 (22-63) | 56.3 (25-63) | 4 ± 20 | 37.5 (25-63) | 37.5 (25-63) | -1 ± 24 | 4.6 (-9 to 18) |
| | **GH** | 20 (5-35) | 20 (15-40) | 5 ± 16 | 20 (15-30) | 25 (10-35) | 1 ± 16 | 3.9 (-5 to13) |
| **SRI** | **RC** | 42.6 ± 17.1 | 56.0 ± 21.6 | 13.5 ± 21.7* | 39.9 ± 8.4 | 50.9 ± 21.7 | 10.9 ± 20.3* | 3.8 (-8 to 15) |
| | **PF** | 28.8 ± 14.5 | 37.6 ± 24.6 | 8.8 ± 22.1 | 33.0 ± 18.2 | 41.8 ± 24.3 | 8.8 ± 24.3 | -1.7 (-15 to 12) |
| | **AS** | 58.8 ± 18.0 | 68.7 ± 16.1 | 9.9 ± 18.9* | 53.3 ± 19.0 | 61.7 ± 21.5 | 8.3 ± 20.0* | 4.3 (-6 to 14) |
| | **SR** | 58.7 ± 17.6 | 60.9 ± 15.8 | 2.2 ± 19.5 | 61.7 ± 13.5 | 67.1 ± 19.3 | 5.4 ± 14.9 | -4.5 (-14 to 5) |
| | **AX** | 44.5 ± 20.1 | 50.3 ± 22.4 | 5.8 ± 28.8 | 45.6 ± 22.5 | 54.2 ± 27.7 | 8.6 ± 27.2 | -3.5 (-18 to 11) |
| | **WB** | 53.4 ± 17.7 | 57.8 ± 18.5 | 4.4 ± 27.1 | 56.6 ± 20.4 | 63.1 ± 21.8 | 6.6 ± 13.5* | -3.7 (-14 to 7) |
| | **SF** | 41.8 ± 16.1 | 45.5 ± 21.5 | 3.7 ± 23.2 | 44.1 ± 23.3 | 52.0 ± 27.1 | 7.9 ± 23.2 | -5.1 (-18 to 8) |
| | **SS** | 46.9 ± 11.7 | 53.8 ± 17.1 | 6.9 ± 19.2 | 47.7 ± 15.6 | 55.8 ± 21.2 | 8.1 ± 17.1* | -1.5 (-12 to 9) |

**Legend Table 3-online:**

Shown are mean SD or #: median (interquartile range (IQR)). #: The MRC and SF-36 scores were skewed distributed; therefore a Mann-Whitney U test was performed to compare the changes within the groups; the delta scores were normally distributed, therefore the changes were compared with a regression analysis. A positive mean difference means a more limited decrease or an increase from baseline to 6 months for the home compared to the in-hospital group. HADS: hospital anxiety and depression scale, consisting of a total score, an anxiety (ANX) and

depression (DEPR) score; CCQ: Clinical COPD Questionnaire, items are divided into three domains: symptoms (symp), functional state (func) and mental state (mental); SF-36: Medical Outcomes Study 36-Item Short-Form Health Survey, consisting of 36 items divided into eight domains: physical functioning (PF), role-physical (RP), role-emotional (RE), bodily pain (BP), mental health (MH), vitality (V), social functioning (SF), and general health (GH); SRI: Severe Respiratory Insufficiency questionnaire, measuring 7 conceptual domains of HRQoL: respiratory complaints (RC), physical functioning (PF), attendant symptoms and sleep (AS), social relationships (SR), anxiety (AX), psychological well-being (WB) and social functioning (SF), and a summary score (SS). [#]Compared to baseline: *: P< 0.05.



Duiverman ML, *et al. Thorax* 2020; 75:244–252. doi: 10.1136/thoraxjnl-2019-213303

**Table 4-online: Exacerbations and hospitalisations**

| | **Home N=30** | | **Hospital N=31** | | **Adjusted mean difference in change Home vs. in-hospital (95% CI)** |
|---|---|---|---|---|---|
| | 6 months prior to inclusion | Study period (6 months after NIV start) | 6 months prior to inclusion | Study period (6 months after NIV start) | before-after mean (95% CI) |
| Exacerbations, n | 2 (1-3) | 1.5 (0-3) | 2 (1-3) | 1 (1-3) | -0.9 (-0.9 to 0.7) |
| Hospitalisations, n | 1 (0-1) | 0 (0-1) | 1 (0-1) | 0 (0-1) | -0.1 (-0.6 to 0.4) |
| Hospital days, n | 5.5 (0-10) | 0 (0-3.5)* | 6 (0-16) | 0 (0-8) | -1.5 (-5 to 2) |

**Legend Table 4:** For the number of exacerbations, hospitalisations and hospital days data were skewed to the right, therefore median (interquartile range (IQR)) is presented. All patients randomised who survived up to 6 months (also after study drop-out) were included for this analysis. For the differences between groups adjusted mean differences in change is presented as these data were normally distributed.

**Home initiation and measurements**

Although the protocol prescribed 2 home visits per patient, eventually 3 (range 2 to 7) home visits were performed per patient. Reasons for additional home visits were technical problems mainly with the transcutaneous measurements, mask problems or poor compliance.

In the home group, median 3 (range 1 to 4)) transcutaneous measurements were performed per patient. At home, there were technical problems mostly with the measurement of PtCO2 or transfer of the data by telemonitoring in 16 patients (50%); 30 of the 88 measurements performed (34%) did not succeed in measuring $PtCO_2$. Four patients did not succeed to perform a transcutaneous measurement at home at all, while in the remaining patients at least one measurement was missing; in 10 patients we did not achieve the minimum of 2 valid measurements. It was decided to repeat the measurement in 10 cases needing an additional home visit. In the hospital group also median 3 (range 1-6) transcutaneous measurements were performed, and we succeeded to get at least 2 valid measurements in all patients.

There were no problems with the transfer of ventilator data, except for 1 patient who lacked a stable home internet connection. Patients gave feedback that they were very satisfied with home NIV initiation.



Duiverman ML, *et al. Thorax* 2020; 75:244–252. doi: 10.1136/thoraxjnl-2019-213303

**Table 5-online:** nocturnal transcutaneous measurements during the initiation period.

| | **Home** | | **Hospital** | | |
|---|---|---|---|---|---|
| | Spontaneous breathing | With NIV at the end of the initiation period | spontaneous breathing | With NIV at the end of the initiation period | **Adjusted mean difference in change home vs. in-hospital (95% CI)** |
| **$PtCO_2$, kPa, mean** | 7.9 ± 1.2 | 6.5 ± 6.6 | 8.2 ± 1.6 | 6.6 ± 0.7 | -0.01 (-0.42 to 0.41) |
| **$PtCO_2$, kPa, max** | 8.9 ± 1.2 | 7.2 ± 0.7 | 9.4 ± 1.8 | 7.4 ± 0.9 | 0.18 (-0.31 to 0.66) |
| **$SaO_2$, %, mean** | 89.9 ± 3.9 | 92.0 ± 2.9 | 91.9 ± 4.7 | 93.5 ± 2.8 | 0.2 (-0.9-1.4) |

**Legend Table 5-online:** Data are shown as mean ± SD. A positive mean difference means an increase ($SaO_2$) or less decrease ($PtCO_2$) for the home compared to the in-hospital group. $PtCO_2$: transcutaneously measured $CO_2$, drift corrected, both the mean value and the maximum over that particular night were recorded. $SaO_2$: oxygen saturation.

**Table 6-online: Outcomes in the per protocol population**

| | Home N=23 | | | Hospital N=25 | | | Adjusted mean difference in change home vs. hospital (95% CI ) |
|---|---|---|---|---|---|---|---|
| | **Baseline** | **3 months** | **6 months** | **Baseline** | **3 months** | **6 months** | **6 months-baseline** |
| **$PaCO_2$, kPa** | 7.3 ± 0.9 | 6.7 ± 0.9* | 6.4 ± 0.8** | 7.4 ± 1.0 | 6.5 ± 0.5* | 6.4 ± 0.6** | 0.07 (-0.3 to 0.4) |
| **$PaO_2$, kPa** | 6.8 ± 1.3 | 7.5 ± 1.5 | 7.6 ± 1.2 | 7.3 ± 1.5 | 8.1 ± 1.4* | 8.0 ± 1.2 | -0.31 (-1.0 to 0.3) |
| **$HCO_3$, mmol/L** | 33.1 ± 3.8 | 30.8 ± 3.2* | 29.8 ± 2.9* | 33.6 ± 4.2 | 30.2 ± 2.1** | 29.7 ± 2.8** | 0.3 (-1.1 to 1.7) |
| **FEV1, L** | 0.60 ± 0.16 | na | 0.63 ± 0.20 | 0.59 ± 0.21 | na | 0.66 ± 0.32* | -0.05 (-0.14 to 0.04) |
| **FVC, L** | 2.19 ± 0.55 | na | 2.23 ± 0.85 | 1.90 ± 0.50 | na | 2.20 ± 0.94* | -0.33 (-0.7 to 0.1) |
| **6MWD** | 179 ± 93 | na | 212 ± 100* | 197 ± 85 | na | 239 ± 82* | -14 (-55 to 27) |
| **CCQ-total** | 3.4 ± 0.8 | na | 2.9 ± 1.0* | 3.3 ± 1.1 | na | 2.9 ± 1.2* | -0.05 (-0.5 to 0.4) |
| **SRI-SS** | 47.7 ± 12 | na | 51.4 ± 13 | 48.2 ± 16 | na | 53.3 ± 21* | -1.4 (-9 to 6) |

**Legend Table 6-online**

Results of the per protocol analysis excluding the 1 patient in the hospital group that stopped his NIV but completed the outcome analysis. $PaCO_2$; partial arterial carbon dioxide pressure; $PaO_2$: partial arterial oxygen pressure; $HCO_3$: bicarbonate; $FEV_1$: forced expiratory volume in 1 second; FVC: forced vital capacity; 6MWD: 6 minute walking distance; CCQ:



Duiverman ML, *et al. Thorax* 2020; 75:244–252. doi: 10.1136/thoraxjnl-2019-213303

Clinical COPD Questionnaire; SRI-Ss: Severe Respiratory Insufficiency questionnaire Summary Scale; na: not applicable. Compared to baseline within the group: *: P< 0.05.



Duiverman ML, *et al. Thorax* 2020; 75:244–252. doi: 10.1136/thoraxjnl-2019-213303

**References**

1 Lang RM, Badano LP, Mor-Avi V, et al. Recommendations for cardiac chamber quantification by echocardiography in adults: an update from the American Society of Echocardiography and the European Association of Cardiovascular Imaging. *Eur Heart J Cardiovasc Imaging* 2015;16:233-70 doi:10.1093/ehjci/jev014 [doi].

2 Galie N, Humbert M, Vachiery JL, et al. 2015 ESC/ERS Guidelines for the diagnosis and treatment of pulmonary hypertension: The Joint Task Force for the Diagnosis and Treatment of Pulmonary Hypertension of the European Society of Cardiology (ESC) and the European Respiratory Society (ERS): Endorsed by: Association for European Paediatric and Congenital Cardiology (AEPC), International Society for Heart and Lung Transplantation (ISHLT). *Eur Heart J* 2016;37:67-119 doi:10.1093/eurheartj/ehv317 [doi].

3 Ware JE,Jr, Sherbourne CD. The MOS 36-item short-form health survey (SF-36). I. Conceptual framework and item selection. *Med Care* 1992;30:473-83.

4 Windisch W, Freidel K, Schucher B, et al. The Severe Respiratory Insufficiency (SRI) Questionnaire: a specific measure of health-related quality of life in patients receiving home mechanical ventilation. *J Clin Epidemiol* 2003;56:752-9 doi:S089543560300088X [pii].

5 van der Molen T, Willemse BW, Schokker S, et al. Development, validity and responsiveness of the Clinical COPD Questionnaire. *Health Qual Life Outcomes* 2003;1:13.

6 Bell ML, Fairclough DL, Fiero MH, et al. Handling missing items in the Hospital Anxiety and Depression Scale (HADS): a simulation study. *BMC Res Notes* 2016;9:479,016-2284-z doi:10.1186/s13104-016-2284-z [doi].

7 Bjelland I, Dahl AA, Haug TT, et al. The validity of the Hospital Anxiety and Depression Scale. An updated literature review. *J Psychosom Res* 2002;52:69-77 doi:S0022399901002963 [pii].

8 Zigmond AS, Snaith RP. The hospital anxiety and depression scale. *Acta Psychiatr Scand* 1983;67:361-70.

9 Mahler DA, Weinberg DH, Wells CK, et al. The measurement of dyspnea. Contents, interobserver agreement, and physiologic correlates of two new clinical indexes. *Chest* 1984;85:751-8.

10 Miller MR, Hankinson J, Brusasco V, et al. Standardisation of spirometry. *Eur Respir J* 2005;26:319-38 doi:26/2/319 [pii].

11 Wanger J, Clausen JL, Coates A, et al. Standardisation of the measurement of lung volumes. *Eur Respir J* 2005;26:511-22 doi:26/3/511 [pii].

12 American Thoracic Society/European Respiratory Society. ATS/ERS Statement on respiratory muscle testing. *Am J Respir Crit Care Med* 2002;166:518-624 doi:10.1164/rccm.166.4.518 [doi].

13 Peeters P, Mets T. The 6-minute walk as an appropriate exercise test in elderly patients with chronic heart failure. *J Gerontol A Biol Sci Med Sci* 1996;51:M147-51.

14 Borg GA. Psychophysical bases of perceived exertion. *Med Sci Sports Exerc* 1982;14:377-81.

15 van Boven JFM, van de Hei SJ, Sadatsafavi M. Making sense of cost-effectiveness analyses in respiratory medicine: a practical guide for non-health economists. *Eur Respir J* 2018 doi:1801816 [pii].

16 Available at: https://english.zorginstituutnederland.nl/publications/reports/2016/06/16/guideline-for-economic-evaluations-in-healthcare.

17 Ponikowski P, Voors AA, Anker SD, et al. 2016 ESC Guidelines for the diagnosis and treatment of acute and chronic heart failure: The Task Force for the diagnosis and treatment of acute and chronic heart failure of the European Society of Cardiology (ESC). Developed with the special contribution of the Heart Failure Association (HFA) of the ESC. *Eur J Heart Fail* 2016;18:891-975 doi:10.1002/ejhf.592 [doi].
